# Do radiomics or diffusion-tensor images provide additional information to predict brain amyloid-beta positivity?

**DOI:** 10.1038/s41598-023-36639-7

**Published:** 2023-06-16

**Authors:** Sungyang Jo, Hyunna Lee, Hyung-Ji Kim, Chong Hyun Suh, Sang Joon Kim, Yoojin Lee, Jee Hoon Roh, Jae-Hong Lee

**Affiliations:** 1 Department of Neurology, Asan Medical Center, University of Ulsan College of Medicine, 88, Olympic-ro 43-gil, Songpa-gu, Seoul, 05505 Republic of Korea; 2 Bigdata Research Center, Asan Institute for Life Science, Asan Medical Center, Seoul, Republic of Korea; 3 Department of Neurology, Uijeongbu Eulji Medical Center, Eulji University School of Medicine, Uijeongbu, Republic of Korea; 4 Department of Radiology and Research Institute of Radiology, Asan Medical Center, University of Ulsan College of Medicine, Seoul, Republic of Korea; 5 Department of Physiology, Korea University College of Medicine, Seoul, Republic of Korea

**Keywords:** Biomarkers, Neurology

## Abstract

The aim of the present study was to predict amyloid-beta positivity using a conventional T1-weighted image, radiomics, and a diffusion-tensor image obtained by magnetic resonance imaging (MRI). We included 186 patients with mild cognitive impairment (MCI) who underwent Florbetaben positron emission tomography (PET), MRI (three-dimensional T1-weighted and diffusion-tensor images), and neuropsychological tests at the Asan Medical Center. We developed a stepwise machine learning algorithm using demographics, T1 MRI features (volume, cortical thickness and radiomics), and diffusion-tensor image to distinguish amyloid-beta positivity on Florbetaben PET. We compared the performance of each algorithm based on the MRI features used. The study population included 72 patients with MCI in the amyloid-beta-negative group and 114 patients with MCI in the amyloid-beta-positive group. The machine learning algorithm using T1 volume performed better than that using only clinical information (mean area under the curve [AUC]: 0.73 vs. 0.69, *p* < 0.001). The machine learning algorithm using T1 volume showed better performance than that using cortical thickness (mean AUC: 0.73 vs. 0.68, *p* < 0.001) or texture (mean AUC: 0.73 vs. 0.71, *p* = 0.002). The performance of the machine learning algorithm using fractional anisotropy in addition to T1 volume was not better than that using T1 volume alone (mean AUC: 0.73 vs. 0.73, *p* = 0.60). Among MRI features, T1 volume was the best predictor of amyloid PET positivity. Radiomics or diffusion-tensor images did not provide additional benefits.

## Introduction

Alzheimer’s disease (AD), the most common neurodegenerative disease, is characterized by progressive cognitive decline. Pathologic hallmarks of AD include amyloid-beta (Aβ) deposition and neurofibrillary tangles, with the deposition of Aβ preceding the onset of clinical symptoms of AD. Therefore, Aβ deposition is associated with the progression of cognitive decline in people with normal cognition^1^.

The current gold standard modalities to detect Aβ-positivity (Aβ +) are amyloid positron emission tomography (PET) and the Aβ level in cerebrospinal fluid. Amyloid PET has disadvantages of high cost and use of radioisotopes, and cerebrospinal fluid tapping is invasive. In contrast, magnetic resonance imaging (MRI) is noninvasive and relatively safe. Therefore, previous studies were aimed at predicting Aβ + using demographics and MRI. Most studies used T1 volume or cortical thickness: In a study investigating patients with amnestic mild cognitive impairment (MCI) using cortical thickness in addition to demographics and the *APOE* genotype increased the prediction of Aβ + (area under the curve receiver operating characteristic curve [AUC]: 0.77 vs. 0.89)^2^. Other studies also showed improved prediction when using T1 volume or cortical thickness^3–5^. A few studies used diffusion tensor image in discriminating AD from controls^6, 7^. Radiomics were also used to discriminate AD from controls^8^, and one study showed that radiomics combined with baseline demographic characteristics showed better performance for Aβ + prediction compared to baseline demographic characteristics alone^9^. These previous studies investigated different MRI features. The aim of the present study was to investigate which MRI features among T1 volume, cortical thickness, texture, and diffusion-tensor image (DTI) features might be the most powerful in predicting Aβ + . We developed a stepwise machine learning algorithm using demographics, T1 MRI features (volume, cortical thickness and radiomics), and diffusion-tensor image to distinguish amyloid-beta positivity on Florbetaben PET.

## Results

Among 186 patients with MCI, 114 patients were in Aβ + groups and 72 patients were in Aβ- groups. The median (interquartile range) age did not differ significantly between Aβ + and Aβ- groups (median: 74.0 vs. 72.0, *p* = 0.25; Table 1). Sex or education level did not differ significantly between the two groups (0.41 ≤ *p* ≤ 0.61). Neuropsychological tests showed that memory scores were lower in the Aβ + group than in the Aβ- group (median [interquartile range] = – 2.3 [− 3.4 − − 1.7] vs. − 1.7 [− 2.1 − − 1.3], *p* < 0.001).

**Table 1:** Baseline demographics between patients with amyloid positivity (Aβ +) and patients without (Aβ–).

	Aβ– (n = 72)	Aβ + (n = 114)	*p*-value
Age, years	74.0 (65.0 − 77.5)	72.0 (64.0 − 77.0)	0.25
Female sex, n (%)	39 (54.2%)	70 (61.4%)	0.41
Education, years	12.0 (6.0 − 15.0)	12.0 (9.0 − 16.0)	0.61
MMSE score	25.0 (23.0 − 28.0)	24.0 (22.0 − 26.0)	0.14
CDR			0.18
0	1 (1.4%)	0 (0.0%)	
0.5	70 (97.2%)	107 (94.7%)	
1	1 (1.4%)	6 (5.3%)	
CDR-SOB	1.5 (1.0 − 2.5)	2.5 (1.5 − 3.0)	0.001
Neuropsychological tests			
Attention	– 0.6 (– 1.2 − 0.2)	– 0.3 (– 0.9 − 0.2)	0.10
Language	– 0.5 (– 1.7 − – 0.0)	– 0.7 (– 1.8 − 0.2)	0.98
Visuospatial	– 0.8 (– 2.0 − 0.2)	– 0.4 (– 2.1 − 0.3)	0.73
Memory	– 1.7 (– 2.1 − – 1.3)	– 2.3 (– 3.4 − – 1.7)	< 0.001
Frontal	– 1.4 (– 2.4 − – 0.5)	-1.2 (– 2.2 − – 0.6)	0.92

*Aβ* amyloid-beta, *MMSE* mini-mental state examination, *CDR* clinical dementia rating, *CDR*-*SOB* clinical dementia rating-sum of boxes.

For step 1, we used demographics to predict amyloid positivity (Table 2). Using logistic regression, discriminating Aβ + using only demographic features (age, sex, and education) resulted in a low AUC (mean AUC = 0.52 ± 0.08; Table 2). Using detailed neuropsychological tests (SNSB scores) in addition to demographics improved the discriminating performance for Aβ + than when using MMSE scores in addition to demographics (mean AUC = 0.69 ± 0.08 vs. 0.58  ±  0.09, *p* < 0.001). Models using SVM and RF also showed similar results to logistic regression model.

**Table 2:** Logistic regression to predict amyloid-beta positivity in patients with mild cognitive impairment using demographics and magnetic resonance image features.

	Logistic regression			Support vector model			Random forest		
	Mean	SD	*p*	Mean	SD	*p*	Mean	SD	*p*
**Step 1 Demographic features**									
Age, sex, education	0.52	0.08	< 0.001	0.51	0.08	< 0.001	0.55	0.08	< 0.001
Age, sex, education + MMSE (total)	0.58	0.09	< 0.001	0.56	0.09	< 0.001	0.64	0.08	0.059
Age, sex, education + SNSB (detailed)	0.69	0.08	Ref	0.69	0.08	Ref	0.66	0.08	Ref
**Step 2 T1-weighted magnetic resonance image**									
Age, sex, education + SNSB (detailed)	0.69	0.08	Ref	0.69	0.08	Ref	0.66	0.08	Ref
Age, sex, education + SNSB (detailed) + T1b (thickness)	0.68	0.09	0.03	0.67	0.09	0.008	0.62	0.09	< 0.001
Age, sex, education + SNSB (detailed) + T1 (texture)	0.71	0.08	0.06	0.71	0.08	0.12	0.70	0.08	< 0.001
Age, sex, education + SNSB (detailed) + T1 (volume)	0.73	0.08	< 0.001	0.73	0.08	< 0.001	0.73	0.08	< 0.001
**Step 3 Diffusion-tensor magnetic resonance image**									
Age, sex, education + SNSB (detailed) + T1 (volume)	0.73	0.08	Ref	0.73	0.08	Ref	0.73	0.08	Ref
Age, sex, education + SNSB (detailed) + T1 (volume) + DTI (FA)	0.73	0.08	0.60	0.73	0.08	0.42	0.73	0.08	0.85
Age, sex, education + SNSB (detailed) + T1 (volume) + DTI (MD)	0.74	0.08	0.20	0.73	0.08	0.20	0.73	0.08	0.64

*SD* standard deviation, *MMSE* mini-mental state examination, *SNSB* Seoul neuropsychological screening battery, *DTI* diffusion-tensor image, *FA* functional anisotropy, *MD* mean diffusivity, *Ref* reference.

For step 2, we used T1 features to predict amyloid positivity (Table 2). Using T1 volume in addition to demographics and detailed neuropsychological tests (SNSB scores) significantly improved Aβ + prediction (mean ± SD, 0.73 ± 0.08 vs. 0.69 ± 0.08, *p* < 0.001; Table 2). However, adding T1 thickness showed slightly lower AUC than the model using only demographics and detailed neuropsychiatric tests (0.69 vs. 0.68, *p* = 0.03). Adding texture did not improve the discriminating performance for Aβ + than by using only demographic features and detailed neuropsychological tests (0.69 vs. 0.71, *p* = 0.06).

Also, the machine learning algorithm using T1 volume showed better performance than that using cortical thickness (mean AUC: 0.73 vs. 0.71, *p* < 0.001) or texture (mean AUC: 0.73 vs. 0.68, *p* = 0.002). The results were similar in the models using SVM and RF.

**Figure 1:**
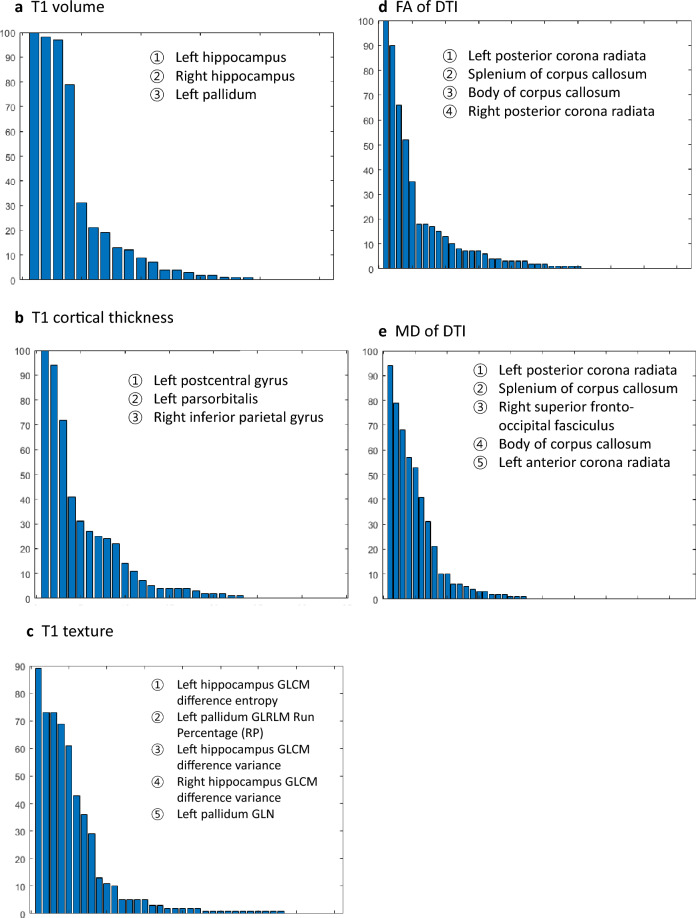
Most selected features from the machine learning process. Bar charts show the most selected features in (**a**) T1 volume, (**b**) T1 cortical thickness, (**c**) T1 texture, (**d**) fractional anisotropy (FA) of diffusion tensor image (DTI), and (**e**) mean diffusivity (MD) of diffusion tensor image (DTI).

The most selected features with > 50% frequency of T1 volume were in the left and right hippocampus and left pallidum (Fig. 1a and Supplementary Table 2). The most selected features with > 50% frequency of T1 cortical thickness were in the left postcentral gyrus, left pars orbitalis, and right inferior parietal gyrus (Fig. 1b). The most selected features with > 50% frequency of texture were in the left hippocampus (GLCM difference entropy and GLCM difference variance), and left pallidum (GLRLM Run Percentage and GLN).

We used FA and MD from DTIs. Adding FA data did not improve the discriminating performance for Aβ + than by using only demographics, detailed neuropsychological test (SNSB scores) results, and T1 volume (mean AUC = 0.73 ± 0.08 vs. 0.73 ± 0.08, *p* = 0.60) (Table 2). Similarly, adding MD data did not improve the discriminating performance (mean AUC = 0.74 ± 0.08 vs. 0.73 ± 0.08, *p* = 0.20), which were similar to the results when using SVM and RF.

The most selected features from DTI with > 50% frequency of FA were in the left posterior corona radiata, splenium and body of the corpus callosum, and right posterior corona radiata (Fig. 1d). The most selected features from DTI with > 50% frequency of MD were in the left posterior corona radiata, splenium and body of the corpus callosum, right superior frontal-occipital fasciculus, and right anterior corona radiata (Fig. 1e).

## Discussion

In this study, T1 volume rather than cortical thickness or texture predicted Aβ + in patients with MCI. Adding DTI features to T1 volume showed no additional benefit in discriminating Aβ + . Therefore, T1 volume in addition to demographics would be the most important features for discriminating Aβ + .

T1 volume rather than cortical thickness or texture showed better discrimination of Aβ + in patients with AD. The most used features of T1 volume were in the bilateral hippocampus and left pallidum. In patients with early-stage AD with mild symptoms, entorhinal volume is reduced by 20–30%, and hippocampal volume is reduced by 15–25%^10^. Therefore, hippocampal volume has been used to detect amyloid deposition or AD^11, 12^. The basal ganglia is also atrophied in AD, and regional atrophic changes in the left caudate nucleus occur with disease progression^13^. Therefore, the hippocampus and globus pallidus volumes as the most used features in the present study were consistent with previous studies. Because the hippocampus was not included in T1 cortical thickness, prediction of Aβ + would have been poor.

Prediction of Aβ + was lower when using T1 texture than when using T1 volume. The texture analysis helps mathematically calculate changes invisible in MRI pixels and is mainly used to find spatial variations in pixel intensity in tissues. Hippocampal radiomic features were suggested as a potential diagnostic biomarker for AD^8, 14, 15^. Some authors hypothesized that the texture analysis could detect the occurrence of neuronal death when the Aβ peptide and neurofibrillary tangles are deposited^16^. It also showed that using both texture and entorhinal cortex volume discriminated patients with MCI from those with AD dementia better than using only hippocampal volume. The distribution and amount of amyloid deposition can vary widely between patients, and it may not be possible to capture this variability using texture analysis alone. The different results between the present study and previous studies might be explained by the fact that we aimed to discriminate Aβ + in patients with MCI with similar disease severity, in which differentiating patients would have been more difficult. Also, age, sex, comorbidities, or sample size that could impact the ability of texture analysis to differentiate patients with amyloid positivity would have affected the results.

The Aβ + prediction rate in the present study was similar to that in previous studies. A 2020 study using demographics, the *APOE* genotype, and neuropsychiatric test results showed an AUC of 0.74, similar to our result of 0.69 when using demographics and neuropsychiatric test results^12^. A 2021 study showed an AUC of 0.68 using demographics, the *APOE* genotype, and neuropsychiatric test and it also showed improved results of 0.78 after adding MRI, not significantly differing from the AUC of 0.73 in the present study^2^.

In a study using only T1 radiomics, the AUC was 0.71, which was similar to the AUC when using demographics, neuropsychiatric tests, and T1 texture in the present study^9^. It showed that the predictive value of Aβ + was low when using cortical thickness (AUC = 0.49) and increased when using T1 and T2 radiomics (AUC = 0.79) in addition to baseline demographics. Consistently, the present study showed that cortical thickness was less predictive compared to T1 texture or volume.

The strength of this study lies in its use of advanced imaging techniques and machine learning algorithms to identify the most important features for discriminating amyloid positivity in patients with MCI, which has important implications for the diagnosis and management of AD. We have several limitations. First, the sample size was relatively small, because we only included patients who underwent MRI, amyloid PET, and detailed neuropsychological tests within one year. Instead, our study population were well-characterized. Second, the study was conducted using a single-center dataset, which may limit the generalizability of the findings to other patient populations and imaging centers. The study would benefit from validation in larger, multi-center datasets. Third, this study only included patients with MCI and did not include healthy controls or participants with subject cognitive impairment. Further research is needed to determine if the findings are applicable to participants with early stage of AD, considering that early intervention would benefit the patients with AD.

In conclusion, T1 volume could predict amyloid deposition in patients with MCI better compared to cortical thickness, T1 texture, or DTI.

## Methods

### Study population

In this retrospective case–control study, we searched the patient registry for patients with MCI who had visited the Memory Clinic at the Asan Medical Center and undergone Florbetaben PET between February 2015 and July 2020. We included 341 patients. Exclusion criteria were: (1) unavailability of MRI data (three-dimensional [3D] T1-weighted and DTI sequences; n = 68); (2) unavailability of neuropsychological test results (n = 21); and (3) interval between the Florbetaben PET, MRI scan and neuropsychological tests exceeding 1 year (n = 66). After applying these exclusion criteria, 186 patients were enrolled. Demographics, including age at neuropsychological tests and MRI, sex, and education level, were obtained. The study protocol was approved by the Institutional Review Board (IRB) of the Asan Medical Center. The study was performed in accordance with relevant guidelines and regulations.

### Neuropsychological tests

All patients underwent detailed neuropsychological tests with the Seoul Neuropsychological Screening Battery (SNSB), in which composite scores of five cognitive domains were included: attention, language, visuospatial, memory, and frontal executive. In the attention domain, we used the digit span forward and backward tests. In the language domain, we used the Boston Naming Test. In the visuospatial domain, we used the Rey–Osterrieth Complex Figure (ROCF) test. In the memory domain, we used immediate recall, delayed recall, and recognition in the Seoul Verbal Learning Test and ROCF test. In the frontal executive domain, we used the phonemic and semantic Controlled Oral Word Association Test results. These raw scores were converted to z-scores for all age and education level groups. The Mini-Mental State Examination (MMSE), Clinical Dementia Rating (CDR), and CDR–Sum of Boxes results were also recorded.

### Amyloid PET acquisition and quantitative measurement

Florbetaben PET images were acquired using the Discovery 690, 710, and 690 Elite PET/computed tomography scanners (GE Healthcare). Amyloid PET images were obtained for 20 min, beginning at 90 min after injecting 300 ± 30 MBq of ^18^F-Florbetaben. Florbetaben PET scans were visually rated as brain amyloid plaque load (BAPL) 1, 2, or 3^17^. We defined Aβ + as BAPL 2 and 3 and Aβ-negativity (Aβ-) as BAPL 1.

### MRI acquisition

Brain MRI was performed with 3D T1-weighted and DTI sequences at the Asan Medical Center using the 3 T MRI scanner (Philips 3 T Achieva; Philips Healthcare). High-resolution structural images were acquired using the 3D T1-weighted sequence with a repetition time of 6.8 ms, an echo time of 3.1 ms, a flip angle of 9°, a field of view of 244 × 244 mm^2^, a voxel size of 1.05 × 1.05 × 2 mm^3^, and a matrix size of 256 × 256 × 100. DTIs were acquired using the single-shot echo-planar imaging sequence with a repetition time of 10,788 ms, an echo time of 70 ms, and a voxel size of 1.2 × 1.05 × 1.05 mm^3^. Diffusion sensitizing gradients were applied along 32 directions with a b-value of 1000 s/mm^2^^18^.

### MRI processing

Each T1-weighted image was processed using the “recon-all” processing stream of the FreeSurfer software (version 6.0; http://surfer.nmr.mgh.harvard.edu). The implemented processing stream included the removal of non-brain tissues, automated Talairach transformation, normalized intensity, tessellated gray/white matter boundary, automated topology correction, and surface deformation. Volumes of 32 regions of interest (ROIs) from aseg atlas^19^ and cortical thicknesses of 68 ROIs from Desikan-Killiany atlas^20^ were used as MRI indicators for T1 volume and cortical thickness, respectively. For the T1 volume, the volume of all ROIs was adjusted to account for the total intracranial volume. The adjustment was performed using the analysis of covariance^21, 22^, such that ${V}_{adj}={V}_{raw}b \left(eTI-\overline{eTIV }\right)$, with *b* being the slope of the linear regression between the volume and eTIV.

We extracted textures from 14 subcortical ROIs (bilateral caudate, putamen, thalamus, pallidum, hippocampus, amygdala, and accumbens) because it was well known that reproducibility of texture features is significantly affected by volume and shape of ROI^23^. The texture features included the intensity, gradient, and texture information, such as gray-level co-occurrence matrices^24–26^, gray-level run-length matrices (GLRLM)^27^, and local binary patterns^28^. A gradient map was built for gradient features, and a local binary pattern map was built for local binary pattern features. Within each ROI, four types of statistical measurements (i.e. mean, standard deviation [SD], skewness, and kurtosis) were each computed from the intensity, gradient, and local binary pattern maps. GLCMs were constructed by systematically considering the relationship between pixel pairs and tabulating the frequency of various gray-level combinations within each ROI. Each element *G*(*i*, *j*) in the gray-level co-occurrence matrices represented the sum of the number of times that a pixel of gray-level *I* occurred in the specified spatial relationship with a pixel of run-length *j*. Similarly, GLRLMs were constructed by evaluating the coarseness of textures in the predetermined direction. A gray level run consisted of a set of consecutive collinear pixels in a certain direction. Each element *R*(*i*, *j*) in the GLRLM indicated the number of runs with pixels of gray-level *I* and run-length *j*. The GLCM and GLRLM were built within the ROI, with 21 statistical measurements computed from the GLCM and 11 from the GLRLM. The source code for the feature extraction is available in part of https://www.github.com/HyunnaLee/StrokeOnset.

DTIs were pre-processed using the Functional Magnetic Resonance Imaging of the Brain (FMRIB) Software Library (FSL; FSL 6.0, University of Oxford, UK; http://www.fmrib.ox.ac.uk/fsl)^29, 30^. First, the raw DTI images were corrected by referencing the B0 volume for eddy current distortions and motion artifacts using the FSL Eddy Correction Tool^31^. The corrected DTI images were stripped to remove non-brain tissues (e.g. skull, muscle) using the FSL Brain Extraction Tool^32^. Second, each image, including the mean fractional anisotropy (FA), mean diffusivity (MD), and eigenvalues λ_1_, λ_2_ and λ_3_, were calculated using the FSL DTI analysis toolkit. MD was then calculated as the mean of the three eigenvalues (λ_1_, λ_2_ and λ_3_). The FA and MD images with the corresponding B0 image were then aligned into the Montreal Neurological Institute 152 Space using the FMRIB’s Nonlinear Image Registration Tool. We used the mean FA and MD of 48 ROIs, which were labeled in the ICBM-DTI-81 white-matter atlas, for DTI indicators^33, 34^. Each MRI features are shown in the Supplementary Table 1.

### Classification and cross-validation

We developed a stepwise machine learning algorithm. As a step 1, we used demographic features including age, sex, education, and neuropsychiatric tests, and chose the best model. For step 2, we added MRI features from T1 image (thickness, texture, and volume) to the best model in step 1. For step 3, we added DTI feature to the best model in step 2.

We used logistic regression (LR) and additional two supervised machine learning algorithms (support vector machine (SVM) and random forest (RF)). We randomly partitioned all enrolled participants into training and test sets using the five-fold cross-validation scheme. In each fold, a small set of features was selected by applying the univariate filter approach, and the selected features were standardized as z-scores using sample mean and SD values from the training sets. The optimal logistic regression model was fitted from the training set. The performance of the optimal model was evaluated by applying features derived from the training set to the test set. These selected features were standardized as z-scores using sample means and SDs from the training sets. The identical test data were evaluated by each machine algorithm to distinguish between Aβ + and Aβ–. Finally, to obtain a more reliable evaluation of the performance, the aforementioned procedure was performed 20 times using a different random partition of the data each time.

Each model’s performance was assessed based on the accuracy, sensitivity, specificity, and AUC. Subsequently, the overall model performance was evaluated based on the mean value of the 100 folds (= five folds × 20 repetitions).

### Statistical analysis

We compared baseline demographic features, and neuropsychological test results between the Aβ + and Aβ- groups, using the χ^2^ test for categorical variables and Student’s *t* test or Kruskal–Wallis test for continuous variables.

## Supplementary Information

Supplementary Table 1.

Supplementary Table 2.

## Data Availability

All deidentified data that support the findings of this study are available upon reasonable request to the corresponding author from other researchers if ethical approval is granted.
